# Molecular dynamics simulations as a guide for modulating small molecule aggregation

**DOI:** 10.1007/s10822-024-00557-1

**Published:** 2024-03-12

**Authors:** Azam Nesabi, Jas Kalayan, Sara Al-Rawashdeh, Mohammad A. Ghattas, Richard A. Bryce

**Affiliations:** 1 Division of Pharmacy and Optometry, School of Health Sciences, Manchester Academic Health Sciences Centre, University of Manchester, Oxford Road, Manchester, M13 9PL UK; 2 Daresbury Laboratory, Science and Technologies Facilities Council (STFC), Keckwick Lane, Daresbury, Warrington, WA4 4AD UK; 3 College of Pharmacy, Al Ain University, Abu Dhabi, UAE

**Keywords:** Molecular dynamics, Self-assembly, Small colloidally aggregating molecules, SCAMs

## Abstract

Small colloidally aggregating molecules (SCAMs) can be problematic for biological assays in drug discovery campaigns. However, the self-associating properties of SCAMs have potential applications in drug delivery and analytical biochemistry. Consequently, the ability to predict the aggregation propensity of a small organic molecule is of considerable interest. Chemoinformatics-based filters such as ChemAGG and Aggregator Advisor offer rapid assessment but are limited by the assay quality and structural diversity of their training set data. Complementary to these tools, we explore here the ability of molecular dynamics (MD) simulations as a physics-based method capable of predicting the aggregation propensity of diverse chemical structures. For a set of 32 molecules, using simulations of 100 ns in explicit solvent, we find a success rate of 97% (one molecule misclassified) as opposed to 75% by Aggregator Advisor and 72% by ChemAGG. These short timescale MD simulations are representative of longer microsecond trajectories and yield an informative spectrum of aggregation propensities across the set of solutes, capturing the dynamic behaviour of weakly aggregating compounds. Implicit solvent simulations using the generalized Born model were less successful in predicting aggregation propensity. MD simulations were also performed to explore structure-aggregation relationships for selected molecules, identifying chemical modifications that reversed the predicted behaviour of a given aggregator/non-aggregator compound. While lower throughput than rapid cheminformatics-based SCAM filters, MD-based prediction of aggregation has potential to be deployed on the scale of focused subsets of moderate size, and, depending on the target application, provide guidance on removing or optimizing a compound’s aggregation propensity.

**Graphical Abstract:**

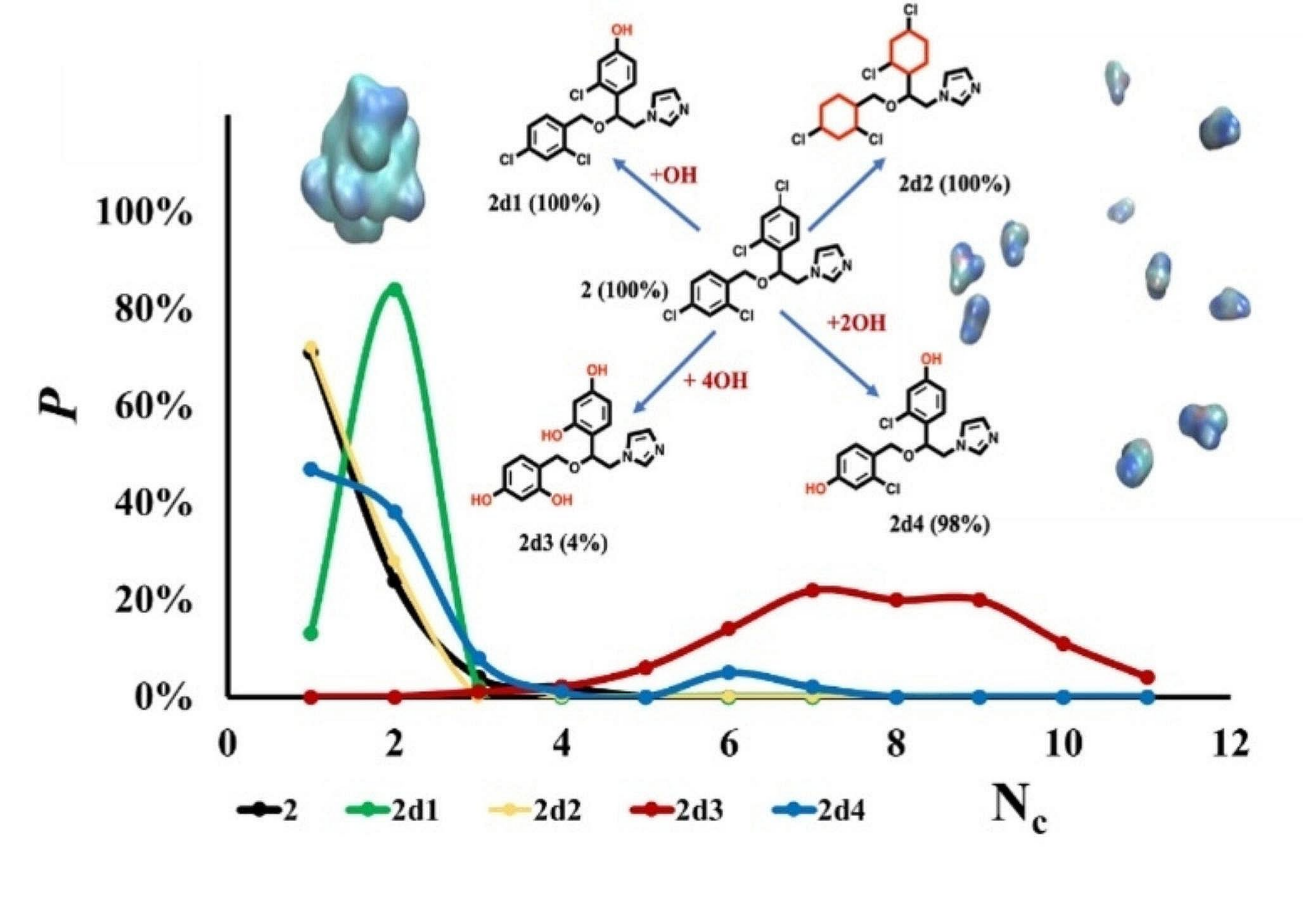

**Supplementary Information:**

The online version contains supplementary material available at 10.1007/s10822-024-00557-1.

## Introduction

The identification of small molecule inhibitors of a target protein from a compound library via high throughput screening (HTS) remains a key tool in the discovery and design of small molecule therapeutics [1–4]. However, HTS campaigns are susceptible to false positive hits, which often arise from organic molecules exhibiting low solubility and a propensity to self-associate, called small colloidally aggregating molecules (SCAMs) [5–7]. The compound assemblies formed by SCAMs are typically on the nanometre to micrometre scale, and interact non-specifically with the protein target to inhibit its function [8]. Recently for example, in cell-based infectivity assays for Covid-19 drug repurposing, 17 of 41 identified candidates displayed artefactual activity due to colloidal aggregation [9]. These colloid aggregates have been shown to exert their nonspecific inhibitory effects by adsorbing and inactivating the enzyme molecules [10–12]. Interestingly, the colloidal properties of small molecule aggregators have also demonstrated potential for exploitation in the formulation field, for example acting as stable vehicles to store enzymes [13]; and as nanoparticle formulations for targeted drug delivery [14, 15].

Therefore, identification of the self-associating properties of small molecules in solution, either as a benefit or a liability, is of high interest. A range of experimental techniques are available to identify such SCAMs – these include NMR [16–18], transmission electron microscopy, fluorescence-based assays and the use of decoy proteins; most commonly, biochemical assays, with and without detergent, and dynamic light scattering (DLS) are used to detect aggregation [1, 5, 10, 11, 19–25], but are somewhat laborious and are typically reserved for the later stages of the drug discovery process. Computational tools to identify potential SCAMs have therefore been attractive prospects for initial screening of large compound libraries. Considerable effort has been invested into developing rapid in silico filters for this purpose: early work by Irwin et al. on the Aggregator Advisor tool [26], a rule-based approach using logP and structural similarity to known SCAMs. Since then, a range of other computational filters, often employing machine learning techniques and larger datasets, have been proposed, including HitDexter [27, 28], SCAM detective [6], BadApple [29], DeepSCAMs [7] and ChemAGG [30]. These tools are rapid and exhibit good accuracy – for example, ChemAGG was able to correctly identify 80% of an external validation set of 5681 aggregators with a prediction probability of greater than 0.9 [30]. However, these empirical tools require good quality datasets for fitting the model, both in terms of assay accuracy, but also spanning sufficient chemical space as required for conducting HTS campaigns of large, diverse chemical libraries.

**Fig. 1:**
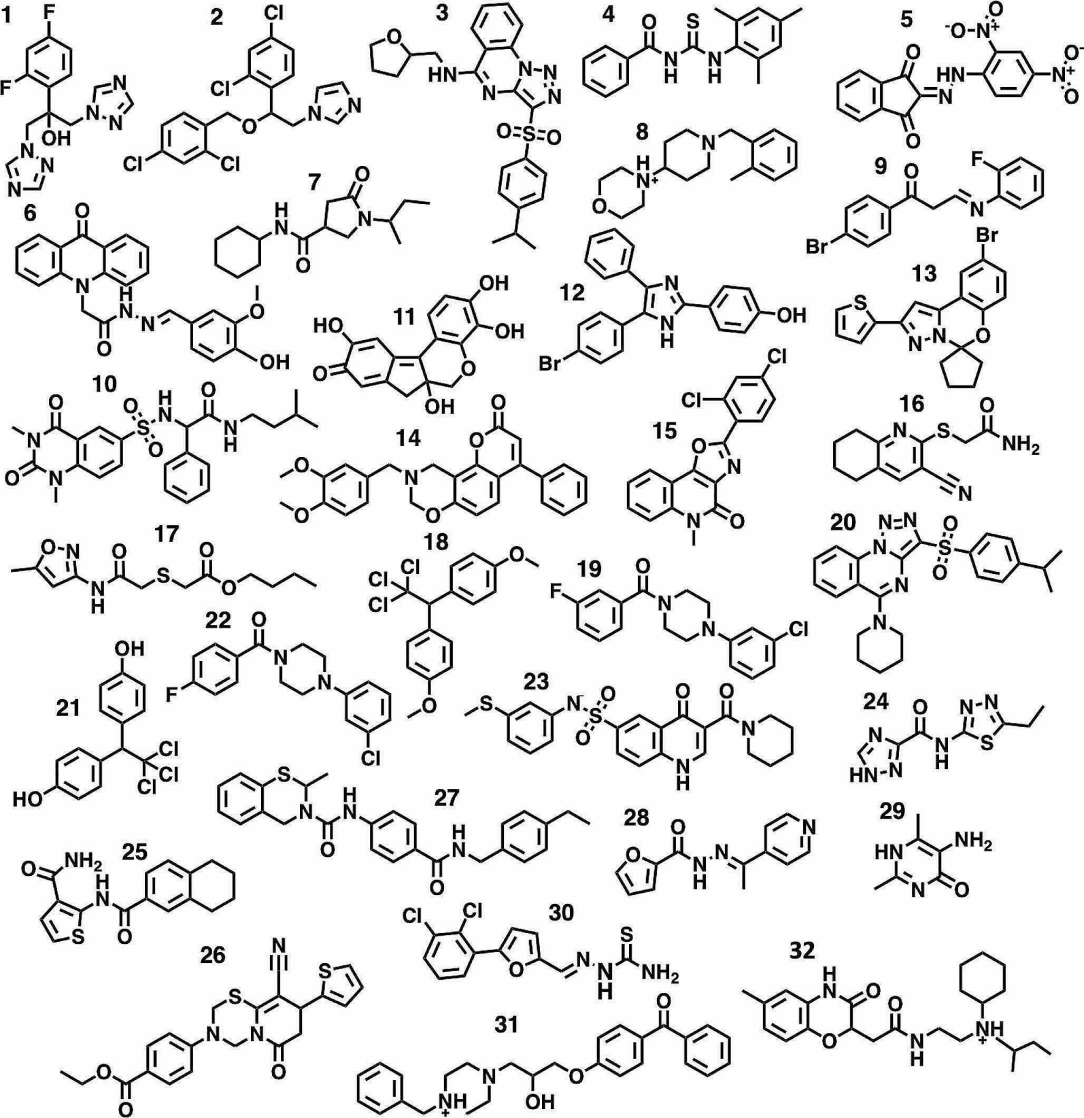
Chemical structure of compounds **1** to **32** studied in this work

In this work, we explore the use of molecular dynamics (MD) simulations as a tool to predict small molecule self-association [12, 31]; the approach does not rely upon fitting to aggregation data for a given chemical space but offers a more fundamental, physics-based route to prediction of aggregation propensity of a small organic molecule in aqueous solution. While considerably more computationally intensive than in silico filters such as Aggregator Advisor and ChemAGG, MD simulations could have potential to complement these approaches, occupying a space in the screening cascade between rapid *in silico* filtering and more time-consuming and expensive experimental characterisation. Of particular value, MD simulations additionally provide detailed information on the scale and dynamics of aggregate formation, the molecular interactions involved and offer potential insight into modifying these interactions to tune self-associating behaviour. In earlier work [12, 31], we found that 100 ns MD simulations were able to distinguish the non-aggregating propensity of fluconazole (**1**, Fig. 1) from the strongly aggregating behaviour of miconazole (**2**, Fig. 1).

Here, we examine the ability of MD simulations to predict aggregation propensity for a larger set of molecules (Fig. 1). In addition to compounds **1** and **2**, we select a structurally diverse set of 30 molecules previously characterised for their aggregation behaviour [1, 19, 26, 30]. These compounds are of varied hydrophobicity, with logP values ranging from − 1.1 (molecule **1**) to 6.1 (molecule **2**), but with the majority in the range 3–4, an intermediate property space where it is often more challenging to predict aggregate formation [26]. After optimising the MD protocol, including a comparison of implicit with explicit solvent models in simulations, we assess the performance of MD assessment in discriminating between known aggregator and non-aggregator compounds, and then apply the approach to derivatives of selected compounds.

## Materials and methods

### System construction

In total, the aggregation behaviour of 32 different compounds (Fig. 1) were simulated in aqueous solution via molecular dynamics. All molecules had been previously assessed experimentally for aggregation properties (for details of assays see Supporting Information Table S1). These compounds were selected based on diversity in chemical structures, and some as being known problem cases for Aggregator Advisor (e.g. **16** and **17**) and ChemAGG filters (e.g. **23** and **28**). Force field parameters for the compounds were assigned using the *antechamber* program [32], according to the general AMBER force field (GAFF2) [33], with AM1-BCC [34] partial charges. Following the approach of Ghattas et al. [31] and balancing system size and computational expense, 11–12 molecules of each solute compound were used to prepare the simulation system, corresponding to millimolar concentrations, well above the micromolar concentrations at which critical aggregation occurs. The molecules were embedded in an octahedral box length of ~ 180 Å containing TIP3P water molecules and neutralised with counterions. 5% v/v of dimethyl sulfoxide (DMSO) and 50 mM of sodium chloride were added to the solvated systems to model experimental assay conditions (Table S2).

### MD simulations

All energy minimization and MD simulations were performed using the AMBER 19 package [35]. Simulations used a time step of 2 fs and the SHAKE [36] algorithm for covalent bonds involving hydrogen atoms. A non-bonded cutoff of 10 Å was used for short-range electrostatic and van der Waals interactions. The PME algorithm for octahedral periodic boundary conditions was used for long-range electrostatic interactions [37]. The solvated systems constructed above were minimized and then heated in two phases using the Langevin thermostat with a coupling constant of 1.0 ps^− 1^ [38]. The first phase was from 0 to 500 K to ensure distribution of solute molecules throughout the simulation box. The second phase was cooling down the system by decreasing the temperature from 500 to 300 K. Each phase was 20 ps in length under NVT conditions. Following this, an equilibration phase of NPT simulation was performed at 300 K and 1 atm for 2 ns. Production simulations were performed for 1 µs at 300 K temperature with structures saved every 20 ps [37]. Clustering analysis was performed using *cpptraj* and an in-house Python program available at https://github.com/jkalayan/ClusterAnalysis. For each analysed simulation frame, solute molecules were grouped into the same cluster if their heavy or hydrogen atoms lie within a given cutoff distance. The intermolecular interatomic distance cutoff for forming a cluster was set to 3.0 Å and the number of molecules in a cluster is given by N_c_. Population distributions as a function of N_c_ used 5000 equispaced frames over MD trajectories. We note that a cutoff value of 3 Å was the minimum to capture correctly the proximity of interacting solutes. A smaller cutoff was found to miss nearest neighbour contacts, whereas cutoffs exceeding 5–6 Å risk including non-interacting molecules into clusters. Potentially one could explore the use of multiple contacts to define direct intermolecular interaction; however, the selection of what number of contacts should be used to group molecules into the same cluster is not well studied and would depend on the shape and flexibility of the molecules involved. By selecting a single interaction within a cutoff to group molecules into clusters, we are able to apply this same qualifier to all molecules studied regardless of the molecule topology. Single molecule descriptors such as LogP, LogD and vsurf_A were calculated using MOE [39].

The above MD protocol was also used for simulations of compound aggregation in implicit solvent. For this, an implementation of the generalized Born neck (GBn) model was used (*igb* = 7), with the *mbondi3* Born radii set [40–44]. The GBn model utilizes a pairwise neck correction term to approximate the dielectric boundary of a molecular surface. This correction removes high dielectric regions smaller than a solvent molecule, effectively eliminating interstitial spaces where a water molecule would be too large to fit. This electrostatic solvation term was used in conjunction with an estimate of the nonpolar contribution to energy and atomic forces via the pairwise solvent-accessible surface area (pwSASA) approach. In the spirit of the study by Huang et al. [45], we explored different surface tension values, finding a value of 0.01 kcal mol^− 1^ Å^−2^ gave the closest agreement with explicit solvent simulations. Lower surface tensions led to very limited self-association across the set of molecules.

## Results and discussion

### Evaluation of MD-based screen for SCAM prediction

To predict the aggregation propensity of known SCAMs and non-aggregators via molecular dynamics simulation, we constructed a dispersed distribution of 11–12 molecules of a given small organic compound of interest throughout the aqueous solution; we then studied the degree to which spontaneous aggregation occurred over the ensuing trajectory. To calibrate our MD protocol, we studied an initial set of compounds, **1–23** (Fig. 1), for one microsecond in explicit solvent. Over the microsecond trajectory of each system, we identified the number of clusters formed by the solute molecules (N_c_).

**Fig. 2:**
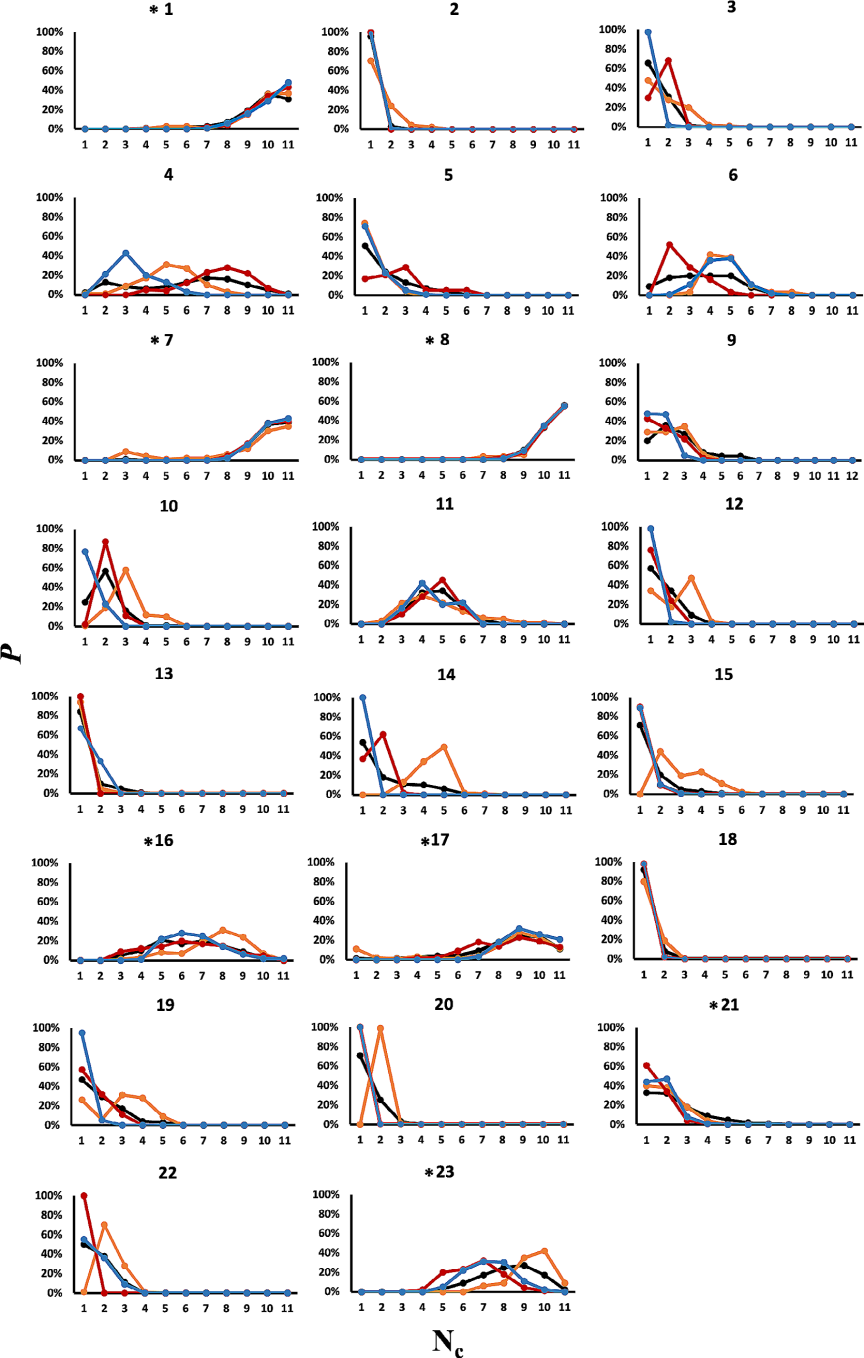
Population *P* (%) of cluster size of molecules **1–23** over 1 µs MD simulation (black) and triplicate MD simulations of 100 ns (orange, red and blue). Compounds that are experimentally observed as non-aggregators were indicated by * next to the molecule label

For a one microsecond simulation of fluconazole **1**, the resulting population distribution in regard to the number of clusters formed, N_c_, indicates that no clustering was observed, such that all eleven molecules remained separated beyond the 3 Å cutoff that defines a cluster (orange, Fig. 2). This is in agreement with experiment and our previous simulations in water boxes of this [31] and larger size [12]. Conversely, for the microsecond simulation of miconazole **2** in solution, strongly aggregating behaviour was exhibited. The distribution of N_c_ has a value of around 1, indicating miconazole molecules aggregate to form mainly one large cluster (Fig. 2); again this is in agreement with experiment and previous simulations [12, 31]. These two compounds illustrate the limiting behaviours of a strong aggregator, having a small N_c_ which approaches 1; and a strong non-aggregator, where N_c_ approaches the number of solute molecules in the simulation. We note that although these simulations were for 11–12 solute molecules in a box of explicitly modelled water, we have previously performed larger scale simulations of **2** in aqueous solution [12]; these displayed very similar behaviour, such that 99 miconazole molecules initially distributed throughout a larger water box also showed significant levels of aggregation [12].

**Fig. 3:**
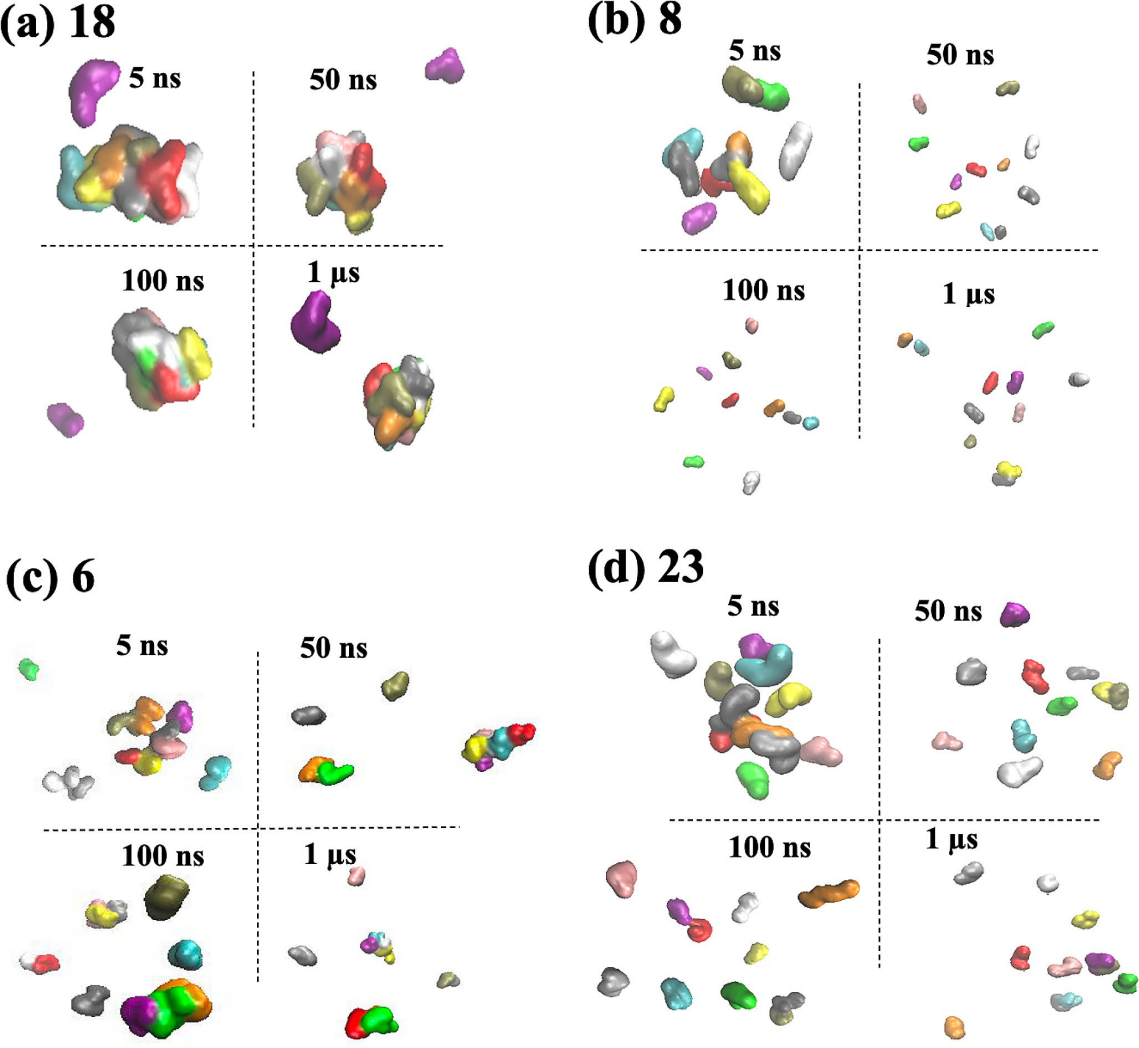
Comparison of MD snapshots of (**a**) molecule **18** (strong aggregator), (**b**) molecule **8** (strong non-aggregator), (**c**) molecule **6** (weak aggregator), and (**d**) molecule **23** (weak non-aggregator); at time slices of 5 ns, 50 ns, 100 ns and 1 µs of MD simulation. Molecules are distinguished by colour

For the remaining molecules **3–23**, a spectrum of aggregation propensity is observed: for example, molecule **18**, the insecticide methoxychlor, forms a persistent large aggregate over the microsecond (Fig. 3a) shown by a population profile that is negatively exponential in shape, similar to **2**. By contrast, molecule **8**, which contains polar piperidine and morpholine rings, shows no propensity to self-associate across the course of the trajectory (Fig. 3b), reflected by a positively exponential N_c_ population profile, similar to compound **1** (Fig. 2).

However, some molecules display intermediate clustering. The population profiles of these molecules do not represent the positive or negative exponential appearance of molecules **1** or **2**, but rather a bell-shaped Gaussian-like form, indicating a spectrum of aggregate sizes are populated over the course of the trajectories. Molecule **6** for instance has a tendency to form aggregates, with a dynamic equilibrium between smaller and larger clusters (Fig. 3c). The dynamic nature of these clusters is also evident from the time dependence of N_c_ (Figure S1) which ranges from around 4–6 clusters initially, to no clusters (at time t = 350 ps) to a completely self-associated system (at t = 630 ps, and again at t = 820 ps). However, the overall tendency of **6** to form fewer larger clusters is clear from the peak in population profile of N_c_ at around 3 (Fig. 2). Interestingly, experimentally molecule **6**, while found to be a SCAM by detergent-based assays, was classified as ambiguous from its scattering behaviour via DLS [1], which may correspond with its simulated intermediate aggregation character. Conversely, molecule **23** exhibits a peak in profile at an N_c_ value of 8 (Fig. 2) which reflects its propensity towards forming a greater number of small clusters (Fig. 3d).

**Fig. 4:**
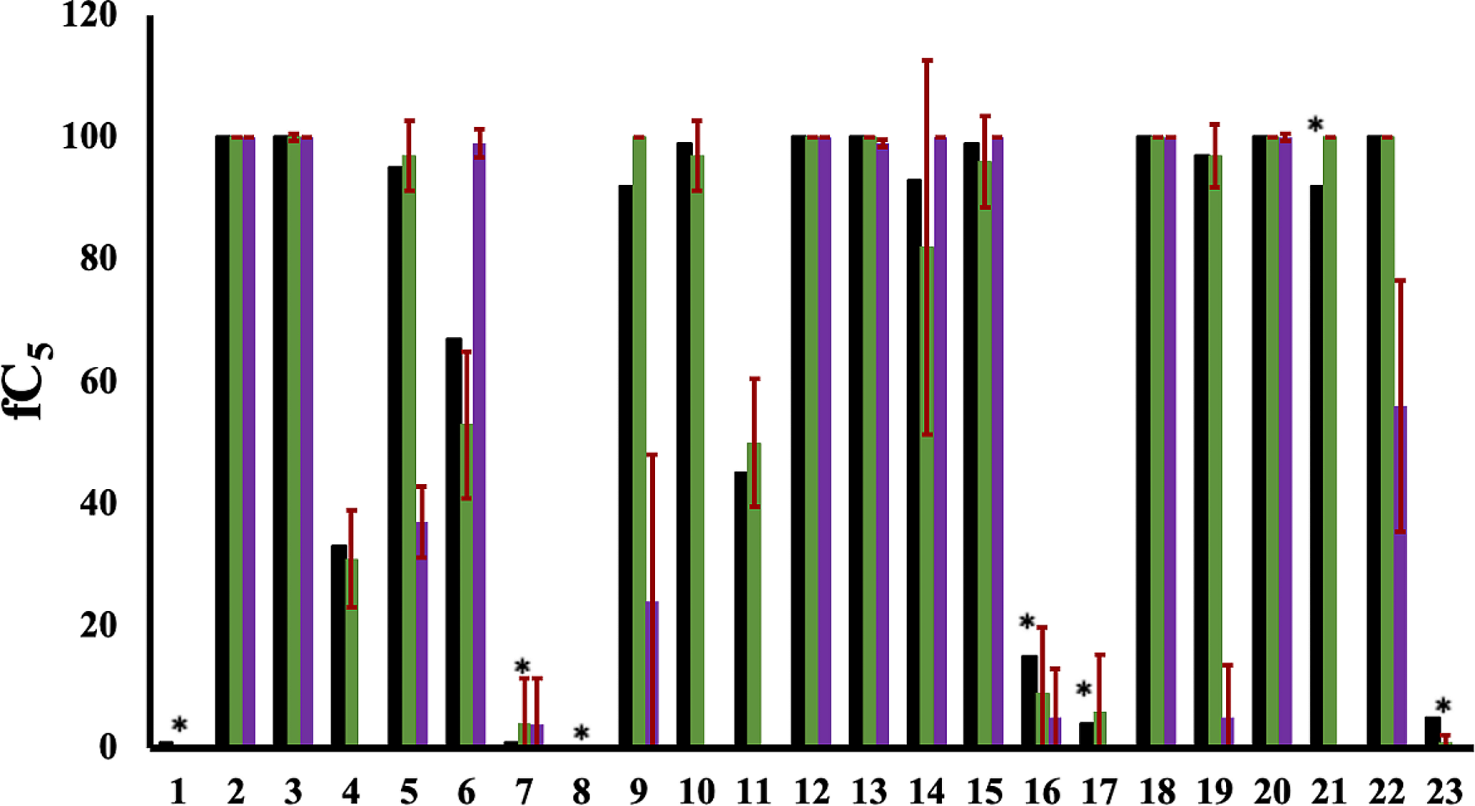
Comparison of calculated fraction of trajectory forming fewer than five clusters, fC_5_, for compounds **1–23** over 1 μs (black) with average of three replicas over 100 ns MD simulation in explicit solvent (green) and implicit solvent (purple). The standard deviation bar of calculated fC_5_ over 100 ns MD simulation, for both explicit and implicit replicas is shown in red. Compounds that are experimentally non-aggregators are signified by the asterisk on top of the related bar

The cluster population profiles are useful in characterizing the differing degrees of predicted propensity for aggregation of a set of molecules from MD simulation. However, for the purpose of providing a parameter with which to filter molecules as SCAM or non-aggregators, we attempt to classify this behaviour towards forming larger clusters using a suitable metric. To do this, we define fC_5_ as the fraction of trajectory in which the molecules form fewer than five clusters over the simulation, i.e. N_c_ <5. Based on fC_5_, we observe 13 of the 17 experimentally determined aggregators have a fC_5_ value close to 100% (Fig. 4).

**Fig. 5:**
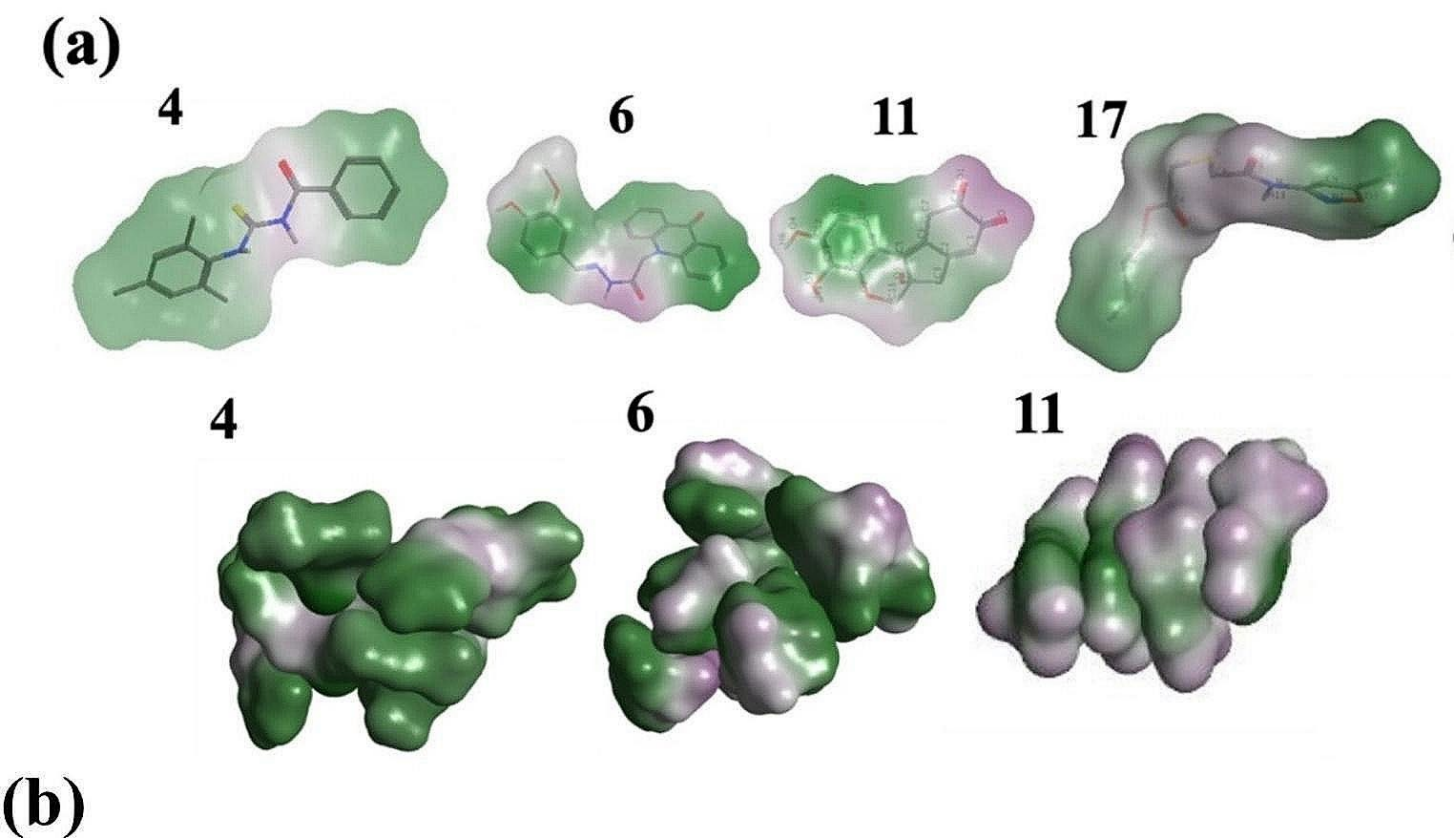
(**a**) Molecular surface for compounds **4**, **6**, **11** and **17**, indicating polar (purple) and nonpolar (green) regions. (**b**) Representative aggregates from microsecond MD trajectories of **4**, **6** and **11**

Known aggregators **4**, **6** and **11**, show more intermediate values of fC_5_, of 36%, 70% and 50% respectively; this reflects their more polydisperse cluster population profiles (Figs. 2 and 4). All three compounds possess a combination of polar and non-polar functionality (Fig. 5a). Analysis of solute-solute interactions indicates close contacts by atoms across the molecular structures (Figure S2). Interestingly, molecule **11**, haematein, has a low logP value of 1.1, lower by 0.6 units compared to similar-sized molecule **17**; the latter is a known non-aggregator which shows very limited self-association during MD simulation (Fig. 2). The total polar surface area (TPSA) of **11** is also larger than **17**, by 26 Å^2^ respectively, yet it seems the greater rigidity of **11** promotes transient stacking (Fig. 5b) which is absent in the more conformationally flexible **17**.

From MD simulations, six molecules have fC_5_ values of less than 20%: **1**, **7**, **8**, **16**, **17** and **23**. All six of these molecules are experimental non-aggregators (indicated by the asterisk in Fig. 4). Molecule **16** shows the highest degree of clustering, with a fC_5_ value of 16% (Fig. 4). In only one case out of the 23 molecule set does MD simulation predict an incorrect outcome: molecule **21** displays strongly aggregating behaviour, with a fC_5_ value of 92%, whereas experimentally it is classed as a non-aggregator. A replicate microsecond trajectory of **21** exhibited a very similar level of aggregation, with a fC_5_ value of 89% (Figure S3). The good agreement between microsecond-length replicate trajectories is further illustrated for the weakly aggregating compound **4** and largely non-aggregating molecule **16** (Figure S3).

These microsecond MD simulations successfully predict aggregation propensity for 96% of the molecules in this sample, which includes molecules such as **17** and **23** which are problematic for computational filters, ChemAGG [30] and Aggregator Advisor [26]. However, a protocol using microsecond simulations is somewhat compute-intensive if applied to a larger set of molecules. Therefore, we examined the predictive ability of 100 ns simulations of **1–23** in explicit solvent, performing three replicates. From the cluster population profiles of these shorter simulations (Fig. 2), while in some cases there is a degree of variation between 100 ns replicates, the trajectories capture rather well the aggregation profiles from 1 µs simulation of the data set. This is also reflected by the close agreement in fC_5_ value across **1–23**, as computed from the 1 µs simulation and the 100 ns replicate average (black and green respectively, Fig. 4): the maximum deviation in fC_5_ estimates is only 10%, found for intermediate aggregator molecule **6**.

Besides reducing the length of MD trajectory, to further increase computational throughput, we also explore the effect of using implicit generalized Born solvent model rather than explicitly modelled water molecules. However, while implicit MD simulations predicted qualitatively similar results to the 1 µs or the 100 ns simulations, there were significant quantitative differences for some molecules (Fig. 4). For example, **4** was predicted to be strongly non-aggregating *via* simulation in implicit solvent, but weakly aggregating from explicit solvent MD, with a fC_5_ value of 33% from the microsecond trajectory and 31% from the 100 ns replica average (Fig. 4). A trend towards decreased aggregation was also reflected for other molecules: for example, molecules **10** and **11** switched from prediction as aggregators *via* simulation in explicit water to being misclassified as non-aggregators; molecule **21**, the only molecule misassigned by explicit solvent simulations, was (correctly) predicted to be a non-aggregator via implicit solvent. Molecule **6** was an exception, predicted as a strong rather than a weak aggregator in implicit solvent. Overall, it seemed implicit solvent underestimated the propensity for hydrophobic self-association. This was despite using a relatively large surface tension parameter of 0.01 kcal/(mol/Å^2^) in the solvent model (see Methods).

**Fig. 6:**
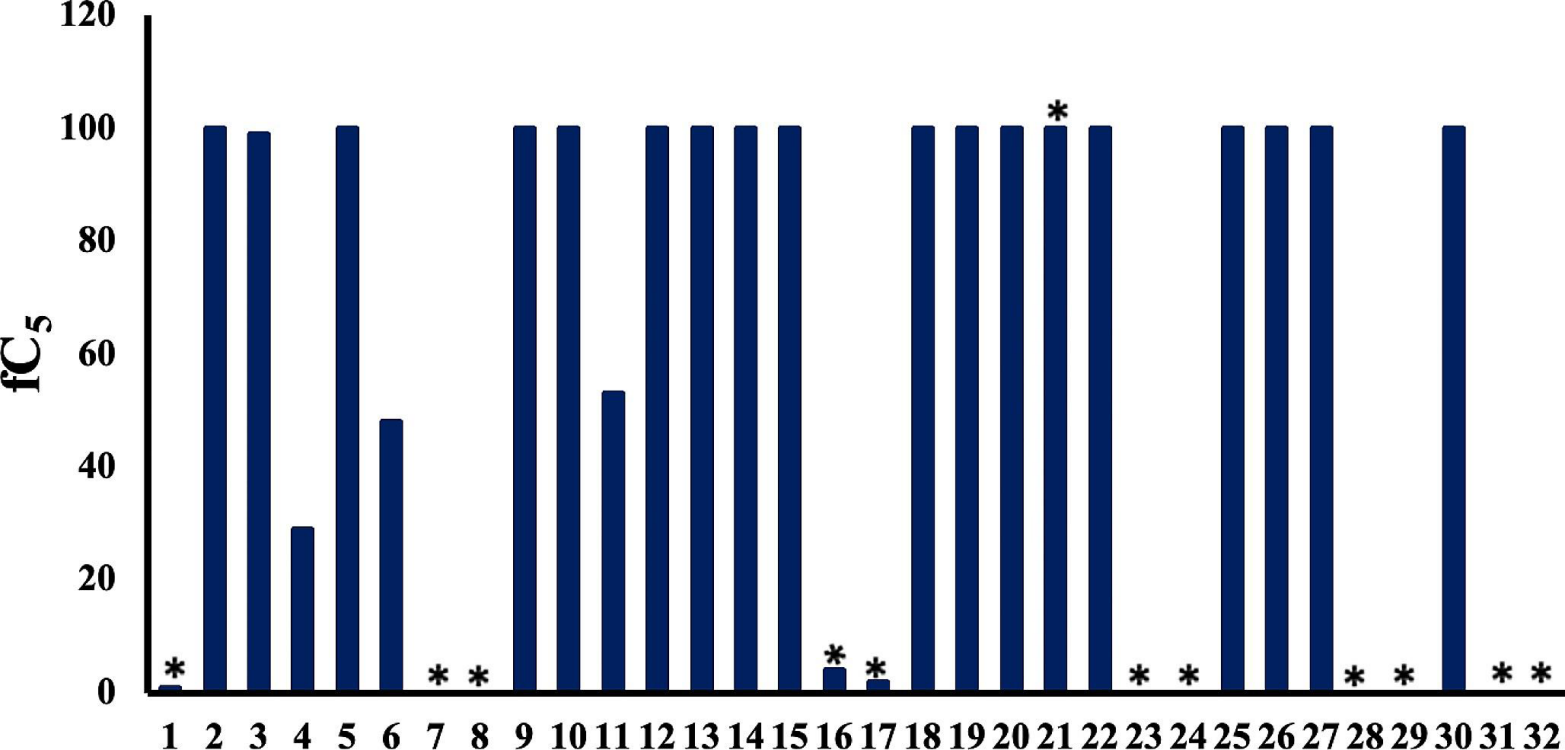
Comparison of calculated fraction of trajectory forming fewer than five clusters, fC_5_, over a 100 ns MD simulation in explicit solvent, for compounds **1**–**32**. Experimental non-aggregators indicated by asterisk

It appears therefore that MD simulations in explicit solvent are required for identifying SCAMs. In a further effort to reduce computational overhead, rather than use triplicate 100 ns simulations, we consider acquisition of only one 100 ns trajectory per molecule. To evaluate this approach, we extend the test set by a further nine molecules, **24–32** (Fig. 1). For the overall set of 32 molecules, the MD-based assessment using a single 100 ns trajectory yields a success rate of 97%, with only compound **21** being misclassified (Fig. 6). Therefore, all 22 molecules of the previous set are correctly classified by the single 100 ns simulation, as well as all nine new cases: MD simulations correctly identify the aggregators **25**, **26**, **27** and **30**, which have fC_5_ values of ~ 100% (Fig. 6); and distinguish these SCAMs from the non-aggregators **24**, **28**, **29**, **31** and **32**, which have fC_5_ values of ~ 0%. Therefore, this success rate of 97% for **1–32** compares with a success rate for the same set of 75% via Aggregator Advisor and 72% via ChemAGG (Table S4).

Indeed, the ChemAGG filter misassigns molecule **11** as a non-aggregator. However, as noted earlier, we can observe from MD simulation the subtle propensity of **11** to self-assemble, such that it forms a range of aggregates of two to four molecules in size (Fig. 5b). By contrast, molecules **31** and **32** are known non-aggregators despite having high computed logP values [39], of 4.0 and 3.6 respectively (Table S3). Aggregator Advisor misclassifies **31** and both ChemAGG and Aggregator Advisor fail for **32**; the MD protocol correctly identifies both as non-aggregators. Indeed, for these cases, the distribution coefficient logD appears to be a more suitable measure than logP, capturing the lower effective hydrophobicity due to ionization, with logD values of 2.8 and 1.3 respectively (Table S3).

We also note in our previous MD study [31] of **1** and **2** that the higher amphiphilic moment of **2**, alongside its greater planarity, reflected the differing aggregation behaviour of these two molecules. We may estimate the amphiphilic moment using the MOE [39] descriptor vsurf_A, which reflects the magnitude of separation between centres of hydrophobic and hydrophilic character in a molecule: miconazole **2** has a vsurf_A value of 6.2 but fluconazole **1** possesses a value of only 3.1. In the set of 32 molecules considered here, there are several known SCAMs with high vsurf_A values, in the range 5–6, namely **2**, **12**, **15**, **16**, **19**, **22** and **30** (Table S3). These are all correctly predicted as SCAMs by MD. However, Aggregator Advisor misclassifies **16**; and ChemAGG misassigns **2**, **12** and **16** (Table S4). Interestingly, compounds **8** and **32** also have high vsurf_A values, of 6.7 and 6.3 respectively (Table S3) but are non-aggregators experimentally. The two *in silico* filters correctly predict **8** as a non-aggregator but misclassify **32**; however, MD predicts the behaviour of both **8** and **32** correctly.

Indeed, the vsurf_A value does not seem to predict well aggregator from non-aggregator for **1**–**32** (Figure S4). Rather, the distribution coefficient logD provides a more discriminative descriptor than vsurf_A or logP, providing rather good agreement with experiment and MD simulation (Figure S4). Indeed, simply assuming aggregation occurs for compounds with a logD > 3 leads to only three compounds in the set being misclassified: compound **11** is misassigned as a non-aggregator, and **21** and **23** as aggregators. Thus, once again, misclassification of **21** is found; we examine this compound further in the following section.

### Structure-aggregation relationships from MD simulation

**Fig. 7:**
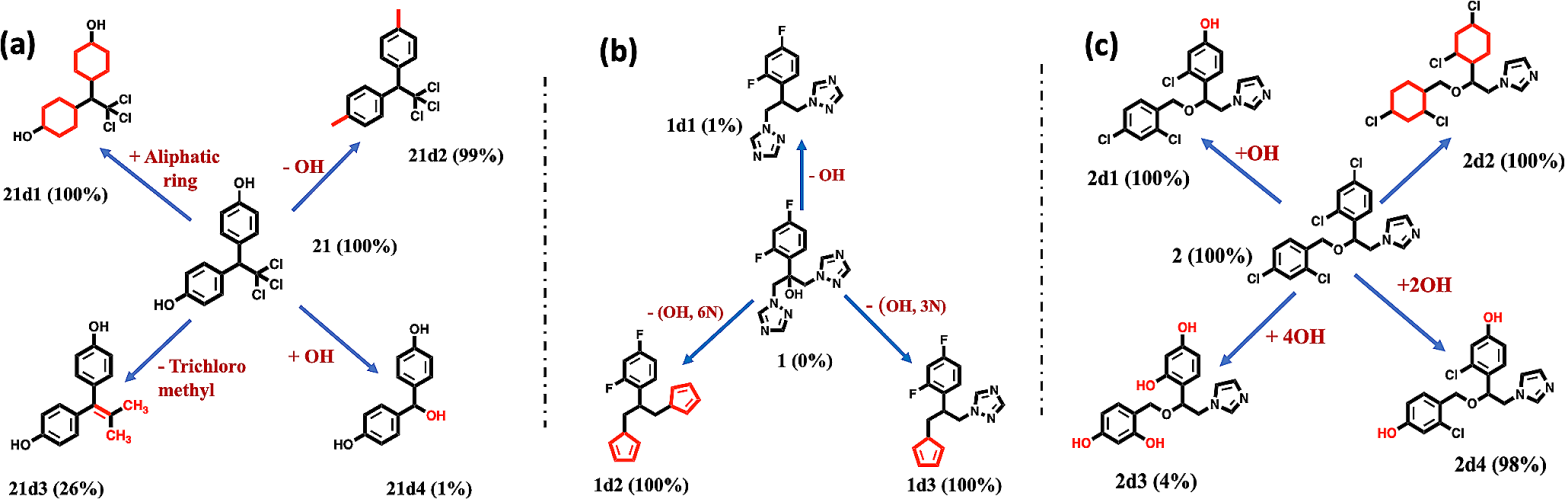
Modification of molecules (**a**) **21**, (**b**) **1** and (**c**) **2** and corresponding cluster population *P* (%) of the compounds and their molecular derivatives as a function of cluster size of 100 ns MD simulations in explicit water. fC_5_ values are given in parentheses

Here, we apply MD to assess the effect of small changes in chemical structure on predicted aggregation propensity. Firstly, to explore further the misclassification of non-aggregator molecule **21**, we conducted a limited *in silico* structure-property analysis of **21**, performing comparative triplicate 100 ns simulations for four closely related derivatives to compute their aggregation propensities. In these simulations, we alter the two phenyl rings of **21** to aliphatic rings (molecule **21d1**, Fig. 7a); or replace the two hydroxyl groups on the phenyl rings with methyl groups (**21d2**). We also change the central part of **21**, replacing the tricholoromethyl group with an alkenyl moiety (**21d3**), or with a hydroxyl function (**21d4**). Interestingly, the modification of the phenyl rings in **21d1** and **21d2** does not lead to a significant change in the strongly aggregating behaviour of **21** (Fig. 7a).

However, replacement of the trichloromethyl group with the alkenyl moiety in **21d3** reduces aggregation from a fC_5_ of 100% to a value of 26% (Fig. 7a). Replacement of the trichloro group by a hydroxyl group in **21d4** leads to the elimination of aggregation (fC_5_ value of 1%). This dramatic switch in simulated behaviour highlights the influence of the largely nonpolar trichloro group on the delicate balance of solute-solute and solute-solvent interactions governing the self-assembly of **21**. As observed earlier, changing from explicit to implicit solvent for **21** also leads to a switch to non-aggregating behaviour, again demonstrating the sensitivity of **21** to changes in intermolecular energetics (Fig. 2). We compare **21d2** with the closely related methoxychlor (**18**): the former only differs from the latter in having two methyl rather than methoxy substituents on the phenyl rings. However, this modest chemical difference is sufficient for MD simulation to predict **21d2** as strongly aggregating and **18**, correctly, as strongly non-aggregating. Given the large influence of small changes in chemical structure, the misclassification of **21** by MD may arise from some subtle imbalance in interaction strengths arising from the force field parameters of the solute and solvent.

As a final point, we note that while self-aggregating molecules can be a nuisance in medicinal chemistry hit identification campaigns, such behaviour may be desirable in generating drug delivery systems for example [15]. To illustrate the potentially useful role MD could play in such processes, using the approach taken for **21** above (Fig. 7), we modify the structures of our archetypal strong non-aggregator **1** and aggregator **2**: in each case, we seek to reverse their self-association behaviour. Given the nature of this computational experiment, we do not consider the synthetic accessibility of these modifications nor present experimental validation. For the case of **1**, removal of the central hydroxyl group did not promote aggregation in MD simulations (**1d1**, Fig. 7b). However, we find that replacement of the nitrogen atoms by carbon in just one of the two 1,2,4-triazole rings in **1** to give **1d3** was sufficient to entirely reverse its predicted behaviour from non-aggregator (fC_5_ of 0%) to aggregator (fC_5_ of 100%). For miconazole **2**, replacement of all four chlorine atoms with hydroxyls led to a switch from aggregator (100%) to non-aggregator **2d3** (4%, Fig. 7c). Other substitutions, for example the switch of only one chloro- substituent for a hydroxyl group (**2d1**) was ineffective in decreasing the self-assembling nature of **2** (Fig. 7c).

## Conclusions

In this work, we assessed the ability of MD simulations to predict the propensity of a set of small organic molecules to self-associate in aqueous solution. From 1 µs simulations, we found a range of aggregation behaviour for the compounds in explicit solvent. Shorter MD simulations of 100 ns provided quantitative agreement with these longer simulations when explicit but not implicit solvent was employed. For the overall set of 32 molecules, using 100 ns MD simulations, we obtained a success rate of 97% as opposed to 75% using Aggregator Advisor and 72% via ChemAGG. The single failure case of the MD method is for a molecule which illustrates a particularly subtle balance between the solute-solute and solute-solvent interactions and may suggest a need to fine-tune parameters of the potentials used. Interestingly, for the 32 molecules of this study, the Aggregator Advisor and ChemAGG filters were outperformed by use of only the distribution coefficient logD, an approach which misclassified only three molecules.

Clearly, while the MD-based approach involves only relatively short trajectories, the protocol remains orders of magnitude slower to acquire than the rapid predictions furnished by filters such as ChemAGG, Aggregator Advisor or logD. However, an MD approach can capture subtle emergent behaviour from complex molecular structures without the need for comprehensive chemically diverse training sets of molecules. Simulations provide detailed information on the scale and dynamics of aggregate formation, the types of noncovalent interactions involved and offer potential insight into modifying these interactions to tune self-associating behaviour. We note that MD simulations using a coarse-grained (CG) potential could offer estimates of aggregation propensity for condensed phase systems of greater size and length scale [46]. However, the sensitivity of aggregation behaviour to small changes in chemical structure that we have observed here, altering the balance of solute and solvent interactions, would provide a significant challenge to CG potentials. Consequently, we suggest atomistic simulations as a useful tool in exhaustive assessment of the aggregation behaviour of small sets of possible SCAMs, for example in optimization of their chemical structures, to either remove or promote aggregation propensity, as pertinent to the target application of the compound.

## Electronic supplementary material

Below is the link to the electronic supplementary material.

Supplementary Material 1

## Data Availability

No datasets were generated or analysed during the current study.
