# Supplementary material for: Molecular dynamics simulations as a guide for modulating small molecule aggregation
Source: J Comput Aided Mol Des. 2024 Mar 12;38(1):11. doi: 10.1007/s10822-024-00557-1 (PMC10933209; doi:10.1007/s10822-024-00557-1)

**Supporting information**

**Molecular dynamics simulations as a guide for modulating small molecule aggregation**

Azam Nesabi^†^, Jas Kalayan^†^, Sara Al-Rawashdeh^†^, Mohammad A. Ghattas^‡^ and Richard A. Bryce*^†^

† Division of Pharmacy and Optometry, School of Health Sciences, Manchester Academic Health Sciences Centre, University of Manchester, Oxford Road, M13 9PL, UK

Science and Technologies Facilities Council (STFC), Daresbury Laboratory, Keckwick Lane, Daresbury, Warrington, WA4 4AD, UK.

‡ College of Pharmacy, Al Ain University, Abu Dhabi, UAE

**Table S1.** Known aggregator (Agg)/non-aggregator (Non-agg) compounds used in this work **1** - **32**, with source of data for experimental assay results and observation of behaviour from MD simulations. Cluster population profiles (Figure 2) are described as exponential shaped distributions for strong aggregators (EXP-) or strong nonaggregators (EXP+); and as Gaussian shaped distribution for weaker aggregation behaviour, which bias towards larger (GAUSS-) or smaller clusters (GAUSS+) or neither (GAUSS).

| **Name** | **Ref.** | **Enzyme/Detergent assay** | **MD simulation/Cluster population profile** |
| --- | --- | --- | --- |
| **1** | Feng et al. | Non-agg | Non-agg / EXP+ |
| **2** | Feng et al. | Agg | Agg / EXP- |
| **3** | Irwin et al. | Agg | Agg / EXP- |
| **4** | Irwin et al. | Agg | Agg / GAUSS- |
| **5** | Feng et al. | Agg | Agg / EXP- |
| **6** | Feng et al. | Agg | Agg / GAUSS- |
| **7** | Feng et al. | Non-agg | Non-agg / EXP+ |
| **8** | Feng et al. | Non-agg | Non-agg / EXP+ |
| **9** | Yang et al. | Agg | Agg / GAUSS- |
| **10** | Yang et al. | Agg | Agg / GAUSS- |
| **11** | Yang et al. | Agg | Agg / GAUSS- |
| **12** | Yang et al. | Agg | Agg / EXP- |
| **13** | Feng et al. | Agg | Agg / EXP- |
| **14** | Yang et al. | Agg | Agg / EXP- |
| **15** | Yang et al. | Agg | Agg / EXP- |
| **16** | Feng et al. | Non-agg | Non-agg / GAUSS |
| **17** | Feng et al. | Non-agg | Non-agg / GAUSS+ |
| **18** | Irwin et al. | Agg | Agg / EXP- |
| **19** | Irwin et al. | Agg | Agg / GAUSS- |
| **20** | Irwin et al. | Agg | Agg / GAUSS - |
| **21** | Irwin et al. | Non-agg | Agg / GAUSS - |
| **22** | Allen et al. | Agg | Agg / EXP- |
| **23** | Feng et al. | Non-agg | Non-agg / GAUSS+ |
| **24** | Feng et al. | Non-agg | Non-agg / GAUSS+ |
| **25** | Yang et al. | Agg | Agg / GAUSS- |
| **26** | Yang et al. | Agg | Agg / EXP- |
| **27** | Yang et al. | Agg | Agg / EXP- |
| **28** | Feng et al. | Non-agg | Non-agg / GAUSS+ |
| **29** | Feng et al. | Non-agg | Non-agg / EXP+ |
| **30** | Yang et al. | Agg | Agg / EXP- |
| **31** | Irwin et al. | Non-agg | Non-agg / GAUSS+ |
| **32** | Irwin et al. | Non-agg | Non-agg / GAUSS+ |

- Feng BY, Shelat A, Doman TN, Guy RK, Shoichet BK. High-throughput assays for promiscuous inhibitors. *Nat Chem Biol*. 2005;1(3):146-148. doi:10.1038/nchembio718
- Irwin JJ, Duan D, Torosyan H, et al. An Aggregation Advisor for Ligand Discovery. *J Med Chem*. 2015;58(17):7076-7087. doi:10.1021/acs.jmedchem.5b01105
- Yang ZY, Yang ZJ, Dong J, et al. Structural Analysis and Identification of Colloidal Aggregators in Drug Discovery. *J Chem Inf Model*. 2019;59(9):3714-3726. doi:10.1021/acs.jcim.9b00541
- Allen SJ, Dower CM, Liu AX, Lumb KJ. Detection of Small‐Molecule Aggregation with High‐Throughput Microplate Biophysical Methods. *Curr Protoc Chem Biol*. 2020;12(1). doi:10.1002/cpch.78

**Table S2.** Molecular details of simulated systems, along with their experimental classification as known aggregator (Agg)/non-aggregator (Non-agg). For each system, the number of solute molecules (Mol), salt, DMSO and water molecules are specified.

| **Mol.** | **Experimental classification** | **Number of** | | | | |
| --- | --- | --- | --- | --- | --- | --- |
|  |  | **Mol.** | $\mathbf{Cl}^{\mathbf{-}}$ | $\mathbf{Na}^{\mathbf{+}}$ | **DMSO** | **Water** |
| **1** | Non-agg | 11 | 32 | 32 | 378 | 34940 |
| **2** | Agg | 11 | 32 | 32 | 783 | 34940 |
| **3** | Agg | 11 | 32 | 32 | 815 | 43088 |
| **4** | Agg | 11 | 32 | 32 | 316 | 34291 |
| **5** | Agg | 11 | 32 | 32 | 239 | 33005 |
| **6** | Agg | 11 | 32 | 32 | 534 | 38781 |
| **7** | Non-agg | 11 | 32 | 32 | 374 | 34925 |
| **8** | Non-agg | 11 | 32 | 32 | 268 | 31882 |
| **9** | Agg | 12 | 32 | 32 | 200 | 31176 |
| **10** | Agg | 11 | 32 | 32 | 762 | 41495 |
| **11** | Agg | 11 | 32 | 32 | 301 | 33509 |
| **12** | Agg | 11 | 32 | 32 | 649 | 39799 |
| **13** | Agg | 11 | 32 | 32 | 307 | 33981 |
| **14** | Agg | 11 | 32 | 32 | 1248 | 48474 |
| **15** | Agg | 11 | 32 | 32 | 378 | 34929 |
| **16** | Non-agg | 11 | 32 | 32 | 304 | 32890 |
| **17** | Non-agg | 11 | 32 | 32 | 294 | 33826 |
| **18** | Agg | 11 | 32 | 32 | 308 | 34146 |
| **19** | Agg | 11 | 32 | 32 | 377 | 34617 |
| **20** | Agg | 11 | 32 | 32 | 631 | 39504 |
| **21** | Non-agg | 11 | 32 | 32 | 311 | 33235 |
| **22** | Non-agg | 11 | 32 | 32 | 401 | 34816 |
| **23** | Non-agg | 11 | 32 | 32 | 857 | 42767 |
| **24** | Non-agg | 11 | 32 | 32 | 76 | 28375 |
| **25** | Agg | 11 | 32 | 32 | 338 | 34951 |
| **26** | Agg | 11 | 32 | 32 | 453 | 36951 |
| **27** | Agg | 11 | 32 | 32 | 412 | 36911 |
| **28** | Non-agg | 11 | 32 | 32 | 126 | 27753 |
| **29** | Non-agg | 11 | 32 | 32 | 62 | 24932 |
| **30** | Agg | 11 | 32 | 32 | 176 | 29845 |
| **31** | Non-agg | 11 | 32 | 32 | 529 | 38843 |
| **32** | Non-agg | 11 | 32 | 32 | 400 | 36505 |

**Table S3.** Comparison of calculated physiochemical properties of compounds **1** – **32** with experimentally measured aggregation. These single-molecule descriptors are computed using MOE (see Methods).

| **Molecule** | **Experimental classification** | **LogP** | **LogD** | **vsurf_A** | **TPSA** | **Mol. Wt.** |
| --- | --- | --- | --- | --- | --- | --- |
| **1** | Non-Aggregator | -1.12 | 1.28 | 3.09 | 81.65 | 306.3 |
| **2** | Aggregator | 6.08 | 6.40 | 6.21 | 27.05 | 416.1 |
| **3** | Aggregator | 3.62 | 4.56 | 2.08 | 98.47 | 451.1 |
| **4** | Aggregator | 3.71 | 4.51 | 2.11 | 73.22 | 298.4 |
| **5** | Aggregator | 4.44 | 3.40 | 0.93 | 150.17 | 340.3 |
| **6** | Aggregator | 4.46 | 4.39 | 1.18 | 91.23 | 401.4 |
| **7** | Non-Aggregator | 1.81 | 1.60 | 1.48 | 49.41 | 266.4 |
| **8** | Non-Aggregator | 2.21 | 1.11 | 6.71 | 16.91 | 261.4 |
| **9** | Aggregator | 4.48 | 4.53 | 1.82 | 29.43 | 320.2 |
| **10** | Aggregator | 2.83 | 3.89 | 3.38 | 115.89 | 472.6 |
| **11** | Aggregator | 1.08 | 1.30 | 1.68 | 107.22 | 300.3 |
| **12** | Aggregator | 5.96 | 5.30 | 5.48 | 48.91 | 391.3 |
| **13** | Aggregator | 5.67 | 3.71 | 4.32 | 27.05 | 389.3 |
| **14** | Aggregator | 5.12 | 5.04 | 2.63 | 57.23 | 430.5 |
| **15** | Aggregator | 3.89 | 5.62 | 5.28 | 46.34 | 345.2 |
| **16** | Non-Aggregator | 1.14 | 1.65 | 5.45 | 79.77 | 247.3 |
| **17** | Non-Aggregator | 1.73 | 1.82 | 1.84 | 81.43 | 286.4 |
| **18** | Aggregator | 5.23 | 5.48 | 3.94 | 18.46 | 345.7 |
| **19** | Aggregator | 3.40 | 4.10 | 5.86 | 23.55 | 318.8 |
| **20** | Aggregator | 4.29 | 5.31 | 4.57 | 80.45 | 435.6 |
| **21** | Non-Aggregator | 4.70 | 4.68 | 4.46 | 40.46 | 317.6 |
| **22** | Aggregator | 3.36 | 4.08 | 5.69 | 23.53 | 318.8 |
| **23** | Non-Aggregator | 2.86 | 3.78 | 2.55 | 95.58 | 457.6 |
| **24** | Non-Aggregator | 0.44 | -0.60 | 2.02 | 96.45 | 224.2 |
| **25** | Aggregator | 2.75 | 3.65 | 2.73 | 72.19 | 300.4 |
| **26** | Aggregator | 3.27 | 4.35 | 2.63 | 73.64 | 425.5 |
| **27** | Aggregator | 5.46 | 5.71 | 3.79 | 61.44 | 445.6 |
| **28** | Non-Aggregator | 1.10 | 2.08 | 3.83 | 67.49 | 229.2 |
| **29** | Non-Aggregator | 0.05 | -0.70 | 3.90 | 67.48 | 139.2 |
| **30** | Aggregator | 5.37 | 3.94 | 6.34 | 95.64 | 314.2 |
| **31** | Non-Aggregator | 3.99 | 2.78 | 3.69 | 54.21 | 431.6 |
| **32** | Non-Aggregator | 3.61 | 1.29 | 6.28 | 71.87 | 402.6 |

**Table S4.** A comparison of MD-based aggregation prediction (MD) of aggregator and non-aggregator (Non-agg) from 100 ns simulations with experiment, Aggregator Advisor and ChemAGG for molecules **1** - **32**. Results that differ from experiment indicated in bold. For ChemAGG, the probability (%) of being an aggregator is also indicated. False positive (FP) defined as known nonaggregator predicted as aggregator; false negative (FN) is the opposite.

| Molecule | Experiment | MD | Aggregator Advisor | ChemAGG |
| --- | --- | --- | --- | --- |
| 1 | Non-agg | Non-agg | Non-agg | Non-agg  2% |
| 2 | Aggregator | Aggregator | Aggregator | **Non-agg**  **4%** |
| 3 | Aggregator | Aggregator | Aggregator | Aggregator  87% |
| 4 | Aggregator | Aggregator | Aggregator | Aggregator  97% |
| 5 | Aggregator | Aggregator | Aggregator | Aggregator  60% |
| $\boldsymbol{6}^{\boldsymbol{a}}$ | Aggregator | Aggregator | Aggregator | Aggregator  98% |
| 7 | Non-agg | Non-agg | Non-agg | Non-agg  20% |
| 8 | Non-agg | Non-agg | Non-agg | Non-agg  12% |
| 9 | Aggregator | Aggregator | Aggregator | Aggregator  91% |
| 10 | Aggregator | Aggregator | Aggregator | Aggregator  84% |
| 11 | Aggregator | Aggregator | Aggregate | **Non-agg**  **17%** |
| 12 | Aggregator | Aggregator | Aggregator | **Non-agg**  **37%** |
| 13 | Aggregator | Aggregator | Aggregator | Aggregator  89% |
| 14 | Aggregator | Aggregator | Aggregator | Aggregator  84% |
| 15 | Aggregator | Aggregator | Aggregator | Aggregator  78% |
| $\mathbf{16}^{\boldsymbol{b}}$ | Non-agg | Non-agg | **Aggregator** | **Aggregator**  **83%** |
| 17 | Non-agg | Non-agg | **Aggregator** | **Aggregator**  **62%** |
| 18 | Aggregator | Aggregator | Aggregator | **Non-agg**  **11%** |
| 19 | Aggregator | Aggregator | Aggregator | Aggregator  78% |
| 20 | Aggregator | Aggregator | Aggregator | Aggregator  95% |
| 21 | Non-agg | **Aggregator** | **Aggregator** | Non-agg  4% |
| 22 | Aggregator | Aggregator | Aggregator | Aggregator  81% |
| 23 | Non-agg | Non-agg | **Aggregator** | **Aggregator**  **78%** |
| 24 | Non-agg | Non-agg | **Aggregator** | Non-agg  36% |
| 25 | Aggregator | Aggregator | Aggregator | Aggregator  60% |
| 26 | Aggregator | Aggregator | Aggregator | Aggregator  64% |
| 27 | Aggregator | Aggregator | Aggregator | Aggregator  96% |
| 28 | Non-agg | Non-agg | **Aggregator** | **Aggregator**  **81%** |
| 29 | Non-agg | Non-agg | Non-agg | Non-agg  2% |
| 30 | Aggregator | Aggregator | Aggregator | Aggregator  84% |
| 31 | Non-agg | Non-agg | **Aggregator** | Non-agg  25% |
| 32 | Non-agg | Non-agg | **Aggregator** | **Aggregator**  **91%** |
| FP | − | 1 | 8 | 5 |
| FN | − | 0 | 0 | 4 |
| FP+FN | _ | 1 | 8 | 9 |
| Percentage True |  | 96% | 75% | 72% |

Figure S1. Time series of the number of clusters formed (<N_c_>) for compounds 1 - 23 over 1 µs; and for 24 – 32 over 300 ns (although for the latter, only the first 100 ns was used in analysis presented in main text). Compounds experimentally observed as non-aggregators indicated by asterisk next to molecule label.


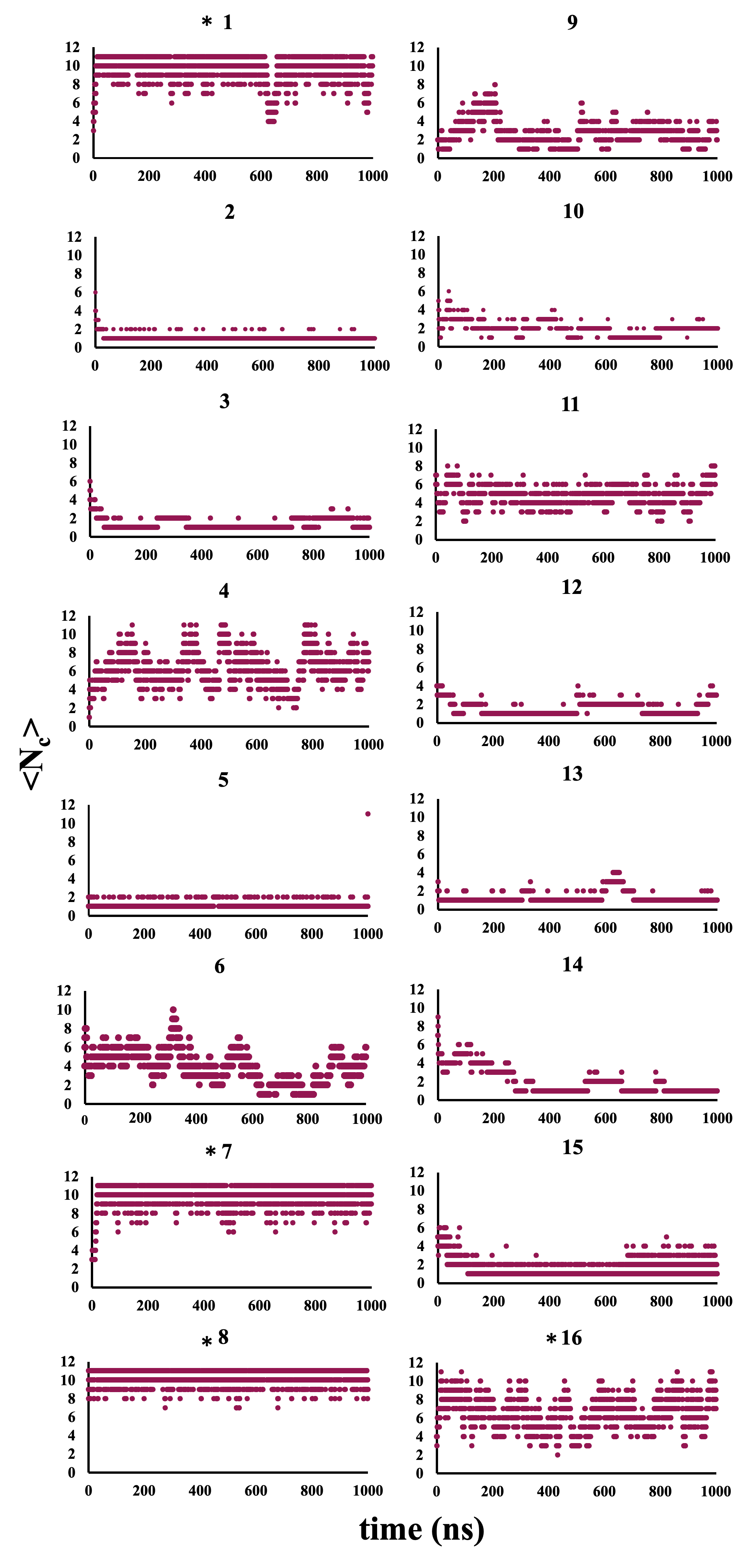


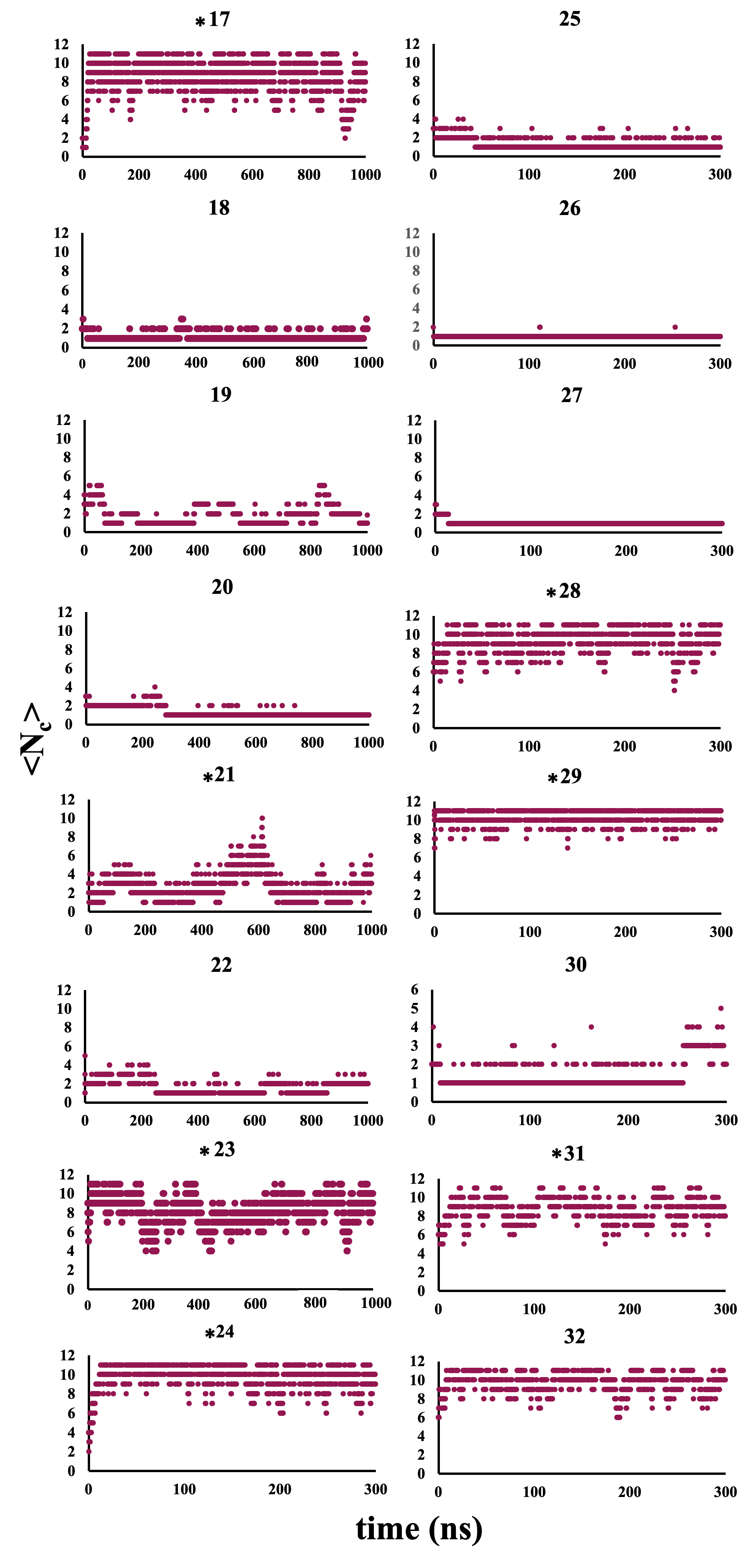


**Figure S2.** Contact analysis for molecules **4**, **6** and **11** over 1 µs of MD simulation, indicating average number of solute-solute interactions <nSS> (in black) and solute-water interactions <nSW> (in blue) for each atom in the compound. Most populated interactions are indicated by bold black atom labels (lefthand chemical structure) or by blue atoms (righthand chemical structure). Central chemical structure has polar (purple) and nonpolar (green) regions indicated. Contacts are defined using an interatomic distance cutoff of 3 Å.


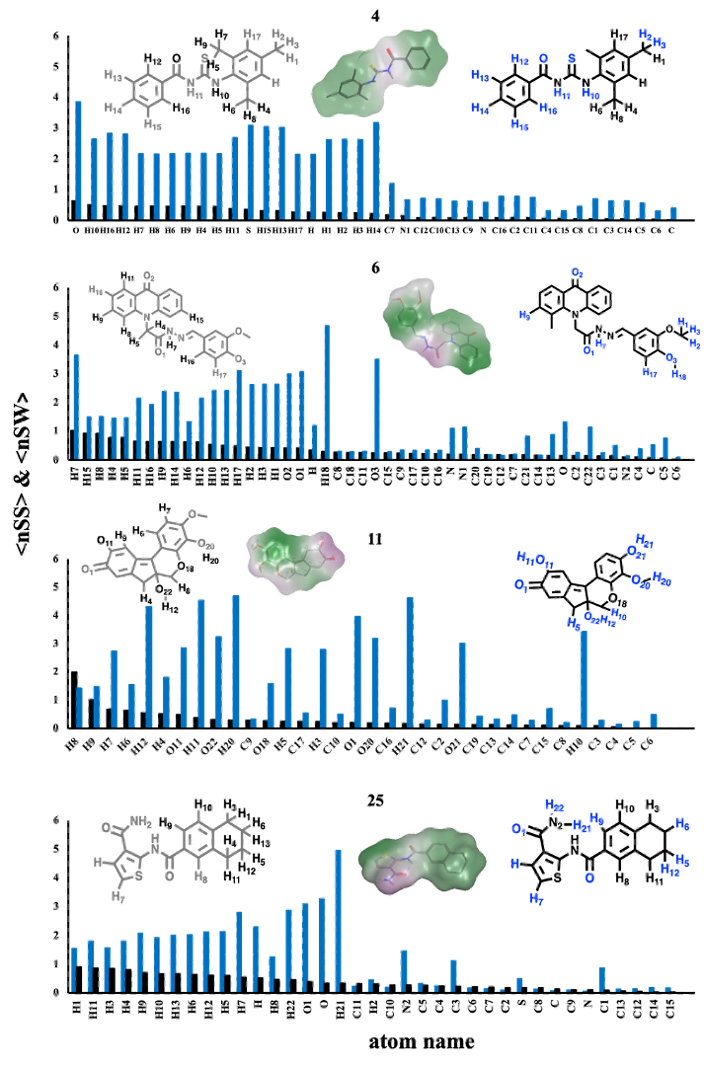


**Figure S3.** Calculated fraction of trajectory forming five or fewer clusters, fC_5_, (%) was computed from two 1 μs MD replicas (black and blue) in explicit solvent, for molecules **4**, **16** and **21**.


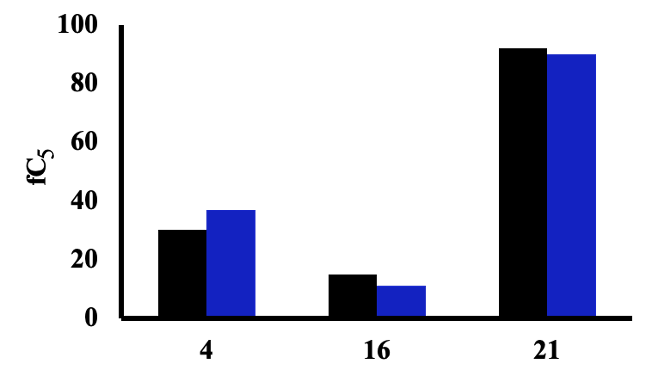


Figure S4. Plot of computed single-molecule (a) LogP, (b) LogD, and (c) vsurf_A values (coloured lines) alongside calculated fraction (%) of trajectory forming five or fewer clusters, fC_5_ (black bars), over a 100 ns MD simulation in explicit solvent, for compounds 1 – 32 (x-axis). Experimental non-aggregators indicated by asterisk.


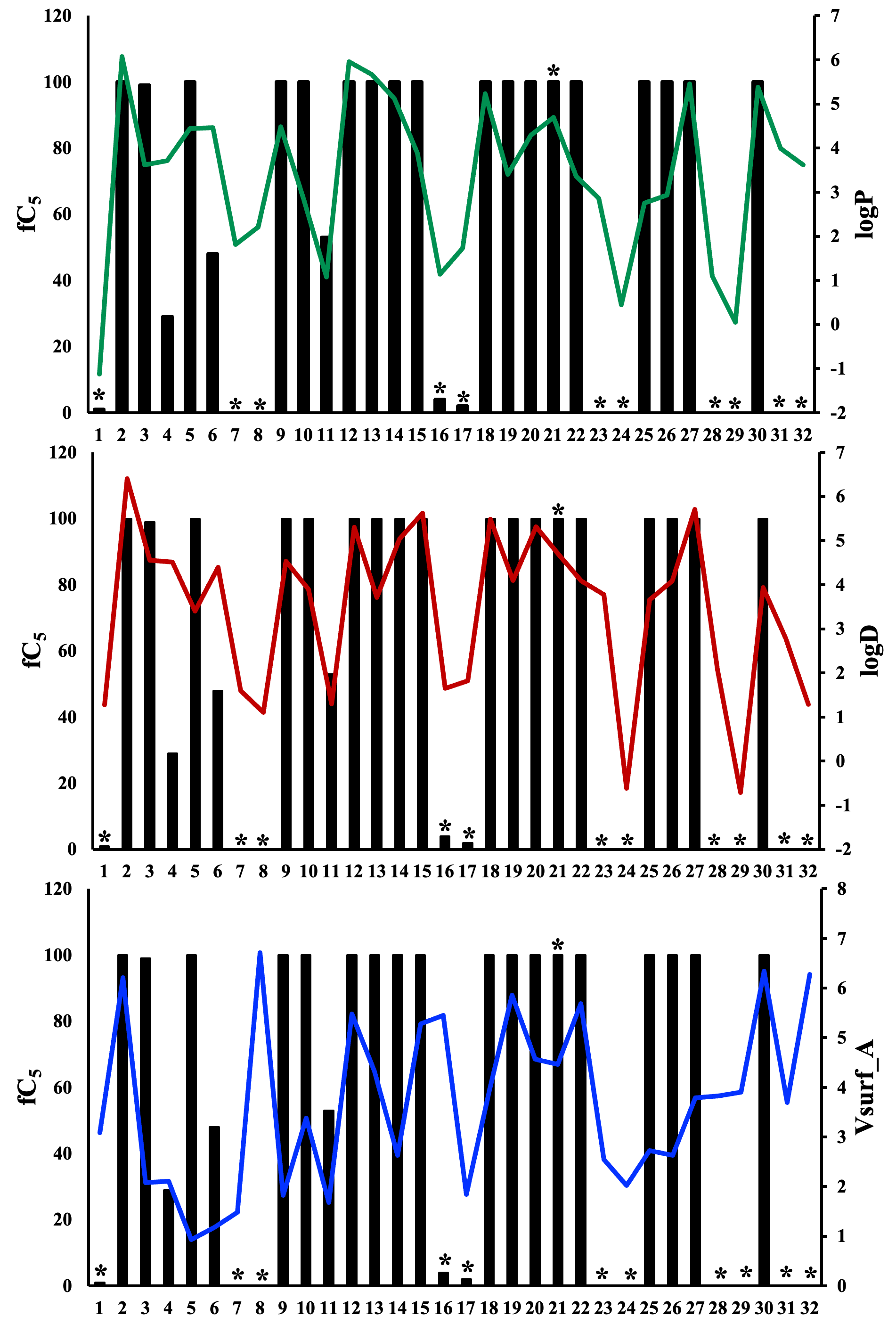

Supplement: Supplementary file 1 — Supplementary Material 1 [file 10822_2024_557_MOESM1_ESM.docx]
